# Acceptability of prophylactic treatment against COVID-19 by paramedics

**DOI:** 10.1192/j.eurpsy.2021.815

**Published:** 2021-08-13

**Authors:** H. El Kefi, K. Kefi, I. Bouzouita, A. Baatout, C. Bencheikh Brahim, W. Krir, A. Oumaya

**Affiliations:** Psychiatric Unit, Military Hospital of Tunis, tunis, Tunisia

**Keywords:** paramedic, therapeutics, coronavirus

## Abstract

**Introduction:**

The year 2020 was marked by the COVID-19 pandemic that killed more than one million people. Scientists around the world are looking for prophylactic treatment against this virus.

**Objectives:**

The objective of our study was to assess the acceptability of prophylactic treatment against COVID-19 by paramedics.

**Methods:**

Descriptive and cross-sectional study including paramedics (nurses, orderlies) from the military hospital of Tunis. Data collection was carried out by a clinical psychologist. We studied the acceptability of prophylactic treatment by paramedics, reasons for refusal and factors that may affect this choice.

**Results:**

A total of 161 paramedics agreed to answer our questionnaire. The average age was 37.73 years. The average number of years worked was 14.95 years. There were 85 women (52.8%) and 76 men (47.2%). Only 59 (36.6%) agreed to take prophylactic treatment for COVID-19. The main reason for refusal was fear of side effects 57 (34.7%). Sufficient hindsight was the main factor that could make them change their decisions. This refusal was definitive for 55 (34.2%) paramedics.

**Conclusions:**

Fear of drug side effects exceeds the fear of COVID.19. An information and communication strategy on the value of prophylactic treatments during a pandemic must be developed.

